# The phosphoproteomic responses of duck (*Cairna moschata*) to classical/novel duck reovirus infections in the spleen tissue

**DOI:** 10.1038/s41598-020-72311-0

**Published:** 2020-09-17

**Authors:** Tao Yun, Jionggang Hua, Weicheng Ye, Zheng Ni, Liu Chen, Cun Zhang

**Affiliations:** Institute of Animal Husbandry and Veterinary Sciences, Zhejiang Academy of Agricultural Sciences, Hangzhou, 310021 China

**Keywords:** Microbiology, Molecular biology

## Abstract

Duck reovirus (DRV) is a fatal member of the genus *Orthoreovirus* in the family Reoviridae. The disease caused by DRV leads to huge economic losses to the duck industry. Post-translational modification is an efficient strategy to enhance the immune responses to virus infection. However, the roles of protein phosphorylation in the responses of ducklings to Classic/Novel DRV (C/NDRV) infections are largely unknown. Using a high-resolution LC–MS/MS integrated to highly sensitive immune-affinity antibody method, phosphoproteomes of *Cairna moschata* spleen tissues under the C/NDRV infections were analyzed, producing a total of 8,504 phosphorylation sites on 2,853 proteins. After normalization with proteomic data, 392 sites on 288 proteins and 484 sites on 342 proteins were significantly changed under the C/NDRV infections, respectively. To characterize the differentially phosphorylated proteins (DPPs), a systematic bioinformatics analyses including Gene Ontology annotation, domain annotation, subcellular localization, and Kyoto Encyclopedia of Genes and Genomes pathway annotation were performed. Two important serine protease system-related proteins, coagulation factor X and fibrinogen α-chain, were identified as phosphorylated proteins, suggesting an involvement of blood coagulation under the C/NDRV infections. Furthermore, 16 proteins involving the intracellular signaling pathways of pattern-recognition receptors were identified as phosphorylated proteins. Changes in the phosphorylation levels of MyD88, NF-κB, RIP1, MDA5 and IRF7 suggested a crucial role of protein phosphorylation in host immune responses of *C. moschata*. Our study provides new insights into the responses of ducklings to the C/NDRV infections at PTM level.

## Introduction

Duck reovirus (DRV) is a fatal aquatic bird pathogen belonging to the genus *Orthoreovirus* in the family Reoviridae^1^. The disease caused by DRV was firstly reported in South Africa in 1950 and isolated in France in 1972 (also called MDRV)^2,3^. In China, infection of DRV, named classical DRV (CDRV), was firstly discovered in Muscovy ducklings in 1997^4^. The disease was characterized by liver and spleen swollen, and covered small white necrotic foci^1,3,5,6^. Additionally, the CDRV infection also causes a series of clinical symptoms, such as basal weakness, diarrhea, hypoevolutism, etc^3,5,7,8^. In the past ten years, a novel duck reovirus (named novel DRV, NDRV) was widely identified in China^7,9–11^. The disease is principally discriminated by hemorrhagic-necrotic lesions in various internal organs, particularly in liver and spleen^7,10–12^. Infection of C/NDRV causes a high morbidity and mortality rate. Although the commercial MDRV vaccine is available, but it is still difficult to prevent their infection and transmission in ducklings within 4 weeks of birth^12^.

The genome and structure of DRV have been reported yet^10,11,13,14^. Completed DRV consists of a double protein capsid shell and 10 double-stranded RNA fragments, including three large size segments, three medium size segments, and four small size segments^10,14^. At least, 10 structural proteins, including three λ translation products, three μ translation products, and four σ translation products, are encoded by the DRV genome^15^. Though the structure of DRV is known, the responsive mechanisms underlying the infection of C/NDRV are uncovered yet.

Innate and adaptive immunity is required for host defense system to fight against pathogen infections. Pattern recognition receptors (PRRs), including Toll-like receptors (TLRs), retinoic acid-inducible gene I (RIG-I)-like receptors (RLRs) and NOD-like receptors (NLRs), play critical roles in initiating the innate and adaptive immune responses^16,17^. By recognizing pathogen-associated molecular patterns (PAMPs), activated PRRs transmit signals to various adaptors, such as Myeloid differentiation primary response gene 88 (MyD88), TIR-domain containing adaptor protein (TRIF), MyD88-adapter-like protein/TIR domain-containing adapter protein (MAL/TIRAP), TRIF-related adaptor molecule (TRAM), and Interferon beta promoter stimulator-1 (IPS-1), which activate a number of downstream kinases (KK complex, MAPKs, TNF receptor-associated factor (TRAF) family member associated NF-κB activator binding kinase 1 (TBK1), and IPS-1 interacts with receptor-interacting protein-1 (RIP-1)) and transcription factors (NF-κB, AP-1, IRF3)^18^. Several previous studies have revealed the responses of intracellular signaling pathways of PRRs to pathogen infections. In Pekin ducks, the expression levels of several PRRs encoding genes, such as*TLR4/7*, *RIG-1* and *Melanoma Differentiation-Associated Gene-5*(*MDA5*), were significantly up-regulated under the infection of duck *hepatitis A* virus^19^. In cherry valley duck, NLRP3, a pattern recognition receptor in host innate immunity, has a certain antibacterial activity during *Escherichia coli* infection^20^. The expression of pattern recognition two receptors encoding genes, *RIG-I* and *MDA5*, was significantly affected by the infection of duck *Tembusu* virus^21^. The interactions between MDRV/NDRV infections and hosts immune response also have been reported in the past years. In MDRV, σB is a structural protein that is able to induce immune response in ducks^22^. A robust immunity response against NDRV in ducklings depends on a subunit vaccine of sigma C protein^12^. Further studies showed that MDRV infection activated host immune response mainly through RIG-I, MDA5 and TLR3-dependent signaling pathways^23^.

The immune responses against pathogen infections play an important role in controlling the prevalence of pathogens.

Recently, increasing studies have focused on screening of C/NDRV infection responsive genes and proteins of ducklings. For examples, a number of genes involved in the RIG-I-like and TLR signaling pathways respond to the infection of MDRV^24^. Several fatty acid degradation-related genes, such as *ATP binding cassette transport G8* and *apolipoprotein A-IV*, were siginificantly induced by the MDRV infection^25^. The expression levels of chemotaxis cytokine receptors encoding genes (*CCR7*, *CCR9*, and *CCR10*) were also significantly up-regulated under the MDRV infection^26^. By two-dimensional polyacrylamide gel electrophoresis, 59 of differentially expressed proteins, involving metabolism and utilization of carbohydrates and nucleotides, anti-stress, and regulation of immune, were detected in Muscovy duck embryo fibroblasts infected with MDRV^27^. Our previous studies showed that proteins involved in the serine protease play roles in the responses to the C/NDRV infections in both liver and spleen^28^. Additionally, two glycolysis-related enzymes, fructose bisphosphate aldolase and pyruvate kinase, were largely up-regulated by the C/NDRV infections^29^.

In addition to transcriptional regulation, post-translational modification (PTM) is an efficient strategy for enhancing the regulation of cellular physiology and the immune responses to virus infections^30,31^. Over the past years, PTMs, such as phosphorylation, ubiquitination, succinylation, acetylation methylation, and crotonylation, have been deeply studied in both prokaryotic and eukaryotic organisms^32–34^. Reversible phosphorylation leads to conformational changes involving various cellular processes, including protein–protein interaction, enzyme inhibition and activation, and energy supply^35^. In mammals, protein phosphorylation is essential for the immune responses to virus infections^36^. For examples, protein phosphorylation is a important component of host cell responses to dengue virus infection^37^. Phosphorylation of eIF2α, an important regulator in the PERK pathway, is essential for the responses to *Chikungunya* and *Sindbis* virus infections^38^. Phosphorylation of transcription factor Sp1 enhances the responses to *herpes simplex* virus type 1 infection^39^. However, the roles of protein phosphorylation in the responses of ducklings to C/NDRV infections are largely unknown.

As a target organ of DRV infection, spleen and live are two major organs of ducklings for digestion and immune responses^28,29^. Our previous studies have identified a number of proteins responsive to the C/NDRV infections at expression level^28,29^. In the present study, the global phosphorylation proteome of duckling spleen was investigated using high-resolution LC–MS/MS integrated to highly sensitive immune-affinity antibody analysis. This study provides new insight into the molecular mechanism underlying the C/NDRV infections in ducklings at the PTM level.

## Materials and methods

### Experimental birds and virus

The experimental infection was carried out using 1-day-old healthy Muscovy ducklings, and their blood samples were checked by RT-PCR. P10, P18 encoding genes was used for the RT-PCR^40^. Twenty seven 1-day-old healthy ducklings were randomly separated into three groups. For the CDRV infection, one group (nine ducklings) was inoculated intramuscular with 0.5 mL of CDRV (ZJ2000M strain, which is the 20th passage virus) at a titer of 10^5.19^ median tissue culture infective dose (TCID_50_) per mL. For the NDRV infection, another group (nine ducklings) was inoculated intramuscular with 0.5 mL of medium infected by NDRV (HN10 strain, which is the 20th passage virus) contained the same TCID_50._ Additionally, one group with nine un-infected ducklings was treated with sterile medium in the same manner. For each treatment group, nine ducklings were divided into three independent subgroups (three ducklings each). Ducklings of each subgroup were euthanatized after 72 h post infection (hpi). Their spleen samples were collected and snap-frozen in liquid N_2_. Subsequently, frozen tissues were kept at − 80 °C until used^28^.

### Protein extraction

The samples were grinded by liquid N_2_ into cell powder and then transferred to a 5 mL centrifuge tube. Four volumes of lysis buffer containing 8 M urea, 1% Protease Inhibitor Cocktail were added to the cell powder, followed by sonication three times on ice using a high intensity ultrasonic processor (Scientz, Ningbo, China). The remaining debris was discarded by centrifugation at 12,000*g* at 4 °C for 10 min. Finally, the supernatant was collected and protein concentration was determined using a bicinchoninic acid assay kit (CW0014; CWBIO, Beijing, China) according to the manufacturer’s instructions^29^.

### Trypsin digestion

For digestion, the protein solution was reduced with 5 mM dithiothreitol for 30 min at 56 °C and alkylated with 11 mM iodoacetamide for 15 min at room temperature in darkness. The protein sample was then diluted by adding 100 mM TEAB to urea concentration less than 2 M. Finally, trypsin was added at 1:50 trypsin-to-protein mass ratio for the first digestion overnight and 1:100 trypsin-to-protein mass ratio for a second 4 h-digestion^28^.

### TMT labeling and HPLC fractionation

After trypsin digestion, peptide was desalted by Strata X C18 SPE column (Phenomenex, Torrance, CA, USA) and vacuum-dried. Peptide was reconstituted in 0.5 M TEAB and processed according to the manufacturer’s protocol for TMT kit. Briefly, one unit of TMT reagent was thawed and reconstituted in acetonitrile. The peptide mixtures were then incubated for 2 h at room temperature, pooled, desalted and dried by vacuum centrifugation. The tryptic peptides were fractionated into fractions by high pH reverse-phase HPLC using Thermo Betasil C18 column (5 μm particles, 10 mm ID, 250 mm length). Briefly, peptides were first separated with a gradient of 8% to 32% acetonitrile (pH 9.0) over 60 min into 60 fractions. Then, the peptides were combined into 6 fractions and dried by vacuum centrifuging^29^.

### Affinity enrichment

Peptide mixtures were first incubated with IMAC microspheres suspension with vibration in loading buffer (50% acetonitrile/6% trifluoroacetic acid). The IMAC microspheres with enriched phosphopeptides were collected by centrifugation, and the supernatant was removed. To remove nonspecifically adsorbed peptides, the IMAC microspheres were washed with solutions of 50% acetonitrile/6% trifluoroacetic acid and 30% acetonitrile/0.1% trifluoroacetic acid, sequentially. The enriched phosphopeptides were eluted from the IMAC microspheres by adding elution buffer containing 10% NH_4_OH. The supernatant containing phosphopeptides was collected and lyophilized for LC–MS/MS analysis^28^.

### LC–MS/MS analysis

The tryptic peptides were dissolved in 0.1% formic acid (solvent A), directly loaded onto a home-made reversed-phase analytical column (15 cm length, 75 μm internal diameter). The gradient was comprised of an increasing from 6 to 23% solvent B (0.1% formic acid in 98% acetonitrile) over 26 min, 23% to 35% in 8 min, climbing to 80% in 3 min, and then holding at 80% for the last 3 min. The experiment was processed at a constant flow rate of 400 nL/min on an EASY-nLC 1,000 UPLC system (Thermo, Shanghai, China).

The peptides were subjected to NSI source followed by MS/MS in Q ExactiveTM Plus (Thermo, Shanghai, China) coupled online to UPLC. The electrospray voltage applied was set at 2.0 kV. The *m/z* scan range was set from 350 to 1,800 for full scan, and intact peptides were detected in the Orbitrap at a resolution of 70,000. Peptides were then selected for MS/MS using NCE setting as 28 and the fragments were detected in the Orbitrap at a resolution of 17,500. A data-dependent procedure that alternated between one MS scan followed by 20 MS/MS scans with 15.0 s dynamic exclusion. Automatic gain control was set at 5E4. Fixed first mass was set as 100 m/z^31^. The MS proteomics data have been set to the ProteomeXchange Consortium by PRIDE partner repository program under identifier PXD016423.

### Database search

The resulting MS/MS data were processed using MaxQuant search engine (v.1.5.2.8). MS/MS data were searched against UniProt database concatenated with reverse decoy database (unip_Anas_8839 database). Trypsin/P was specified as cleavage enzyme allowing up to 4 missing cleavages. The mass tolerance for precursor ions was set as 20 ppm in first search and 5 ppm in main search, and the mass tolerance for fragment ions was set as 0.02 Da. Carbamidomethyl on Cys was specified as fixed modification and Acetylation modification and oxidation on Met were specified as variable modifications. FDR was adjusted to < 1% and minimum score for modified peptides was set > 40^29^.

### Peptide annotation

All identified proteins were annotated searching against Gene Ontology (GO), Kyoto Encyclopedia of Genes and Genomes (KEGG)^41,42^, and InterPro domain databases. GO annotation was derived from the UniProt-GOA database (https://www.ebi.ac.uk/GOA/). All identified peptide IDs were converted into UniProt IDs and then mapped onto the GO database. KEGG was used to annotate the proteins involving in pathways. KEGG online service tool KAAS was used to provide the KEGG description of proteins. KEGG online service tool KEGG mapper was used to map the annotation results on the KEGG pathway database. Identified proteins domain functional description was annotated by InterProScan using protein sequence alignment method^29^.

### Functional enrichment

For each GO, KEGG, and domain category, a two-tailed Fisher’s exact test was employed to test the enrichment of the differentially modified protein against all identified proteins. The GO term, KEGG pathway and domain with a corrected *P* value < 0.05 are considered significant.

For enrichment based clustering, all the categories after enrichment along with their *P* values. The filtered *P* value matrix was transformed by the function x =  − log10 (*P* value). Finally, these x values were z-transformed for each functional category. These z scores were then clustered by one-way hierarchical clustering (Euclidean distance, average linkage clustering) in Genesis. Cluster membership were visualized by a heat map using the “heatmap.2” function from the “gplots” R-package^28^.

### Motif analysis

Motif-x algorithm was used to analysis the model of sequences constituted with amino acids in specific positions of modify-13-mers (6 amino acids upstream and downstream of the site) in all protein sequences. And all the protein sequences in database were used as background parameter. Minimum number of occurrences was set to 20. Emulate original motif-x was ticked, and other parameters with default^34^.

### Statistical analysis

Significant differences in phosphorylation level between two groups were calculated using a one-way analysis of variance with a Tukey's test. Three biological replicates were applied in protein phosphorylation level analysis.

### Ethics statement

Animal experiments were performed in the Institute of Animal Husbandry and Veterinary Sciences (IAHV), Zhejiang Academy of Agricultural Sciences (ZAAS). All ducks used in the present experiments were treated in accordance with the Regulations of the Administration of Affairs Concerning Experimental Animals approved by the State Council of China. The bird protocol used in this study was approved by the Research Ethics Committee of ZAAS (permit number: ZAAS20160802)^28^.

## Results

### Overview of the phosphoproteomic data

**Figure 1:**
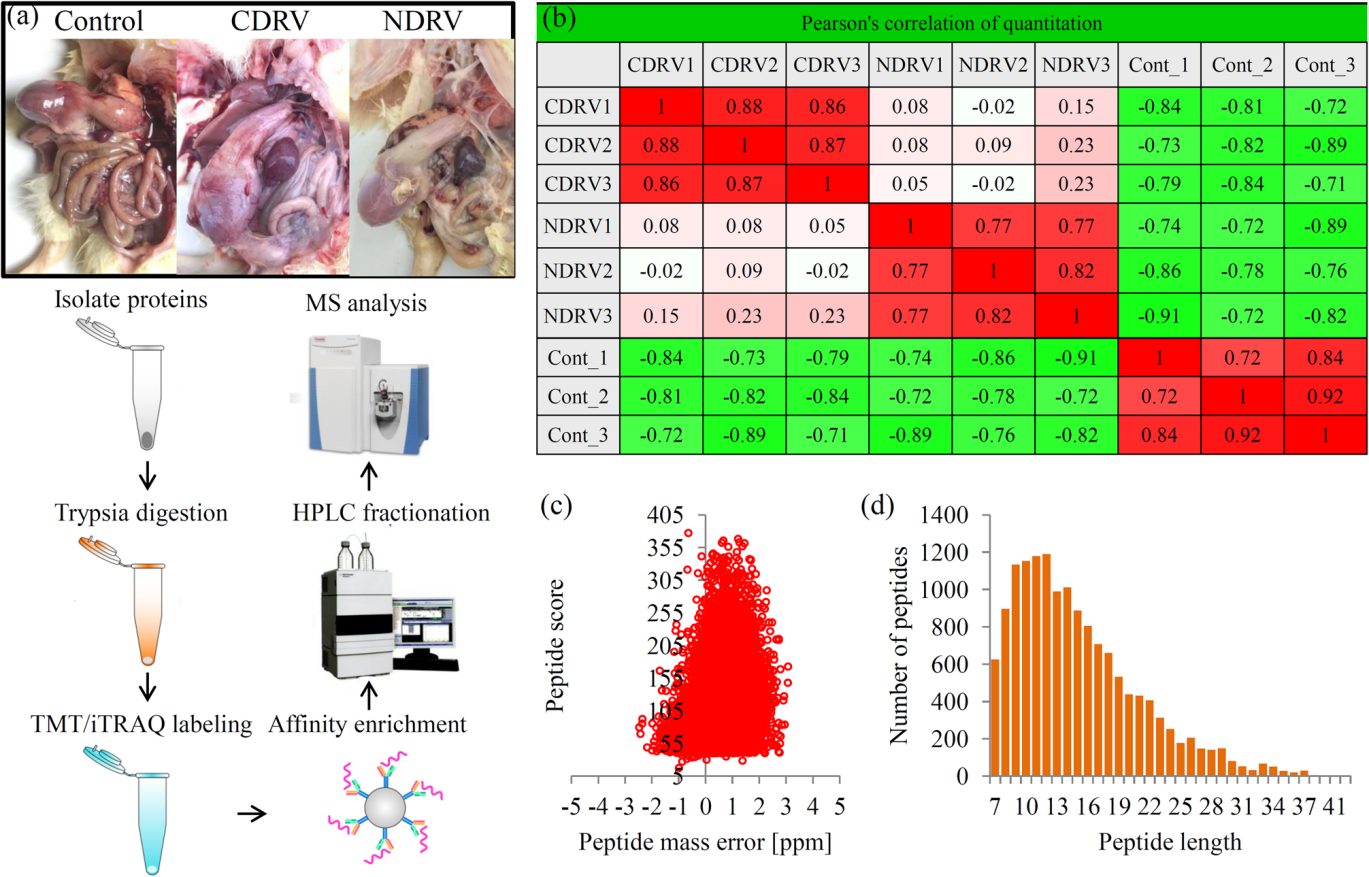
Overview of the phosphorylation proteomes. (**a**) The pictures showed the spleen tissues under control and C/NDRV infections. Experimental strategy for the quantitative analysis of phosphorylation proteomes from three different treatment groups. (**b**) Pearson’s correlation of the phosphorylation proteomes from different sample groups (three biological replicates for each group). (**c**) Mass delta of all identified peptides. X- axis: Peptide Score; Y-axis: Peptides mass delta. (**d**) Length distribution of all identified peptides. X-axis: No. of Peptide; Y-axis: Peptide length.

Through affinity enrichment integrated with LC–MS/MS approach, the phosphoproteomic changes in the spleen tissues of duckling under the C/NDRV infections were investigated (Fig. 1a). Correlation coefficients of sample groups (control and two infected groups) showed good repeatabilities (Fig. 1b). To validate the quality of the MS data, two quality parameters, peptide mass error and peptide length, were analyzed. The distribution of the mass error was less than 0.02 Da and the lengths of phosphopeptides varied from 7 to 20 amino acids, suggesting that the sample preparation met the standards (Fig. 1c,d). A total of 8,504 phosphorylation sites were identified on 2,853 proteins, of which 6,343 sites of 2,407 proteins were quantified (Table S1).

### Analysis of phosphorylation sites

**Figure 2:**
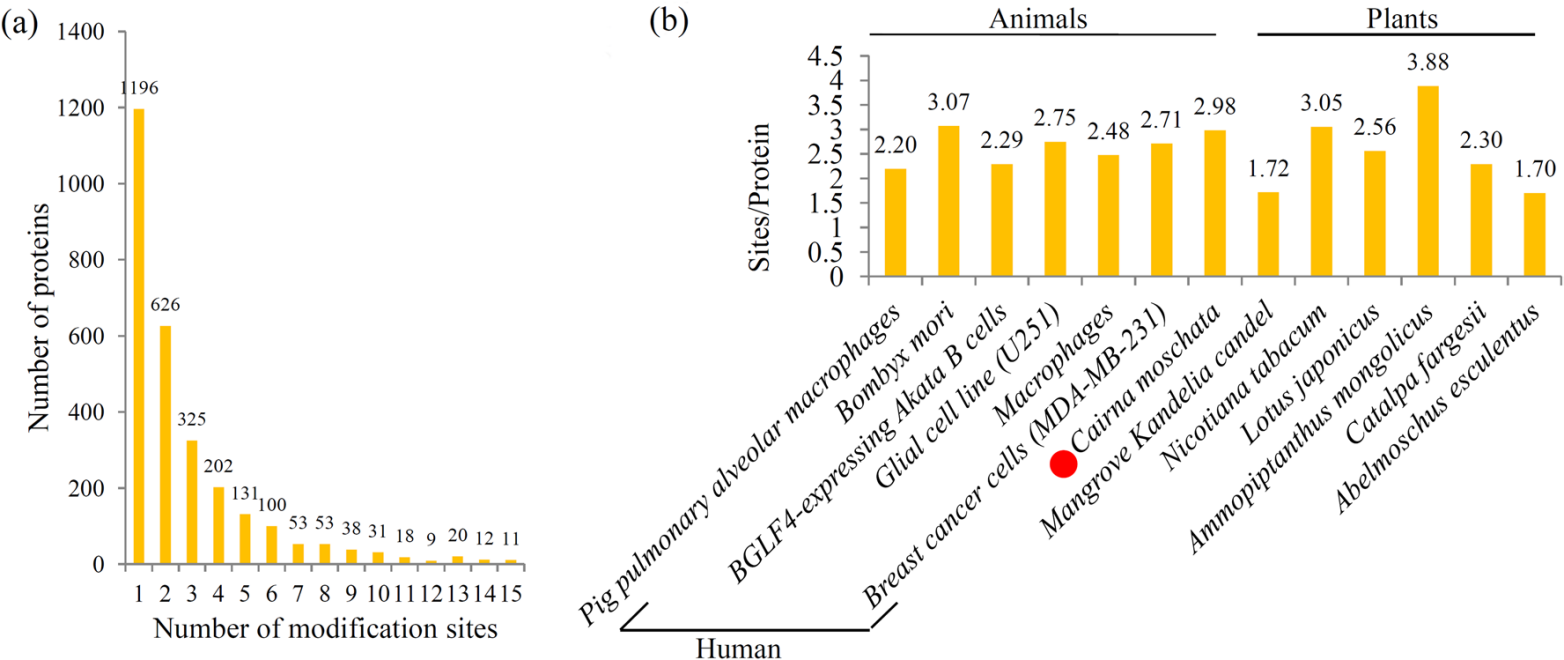
Analysis of the density of phosphorylation sites. (**a**) Distribution of the phosphorylation sites in all identified peptides. (**b**) Comparison of the average densities of phosphorylation sites per protein among various species.

In the spleen tissues of ducklings, 1,196 (41.2%) phosphorylated proteins were modified at a single site, 624 (21.9%) phosphorylated proteins were modified at two sites, and 1,031 (36.1%) phosphorylated proteins contain three and more sites (Fig. 2a). Interestingly, several proteins contain a large number of phosphorylation sites. For examples, there are 36 phosphorylation sites in a chromatin remodeler protein (U3IFN6), 47 phosphorylation sites in a zinc finger CCCH-type protein (U3I4J8), and 83 phosphorylation sites in a serine and arginine repetitive matrix protein (U3I390) (Table S1).

To analyze the density of phosphorylation sites per protein, the phospho-proteome of Muscovy duckling was compared with those published phospho-proteomes in other species. The average number of phosphorylation sites per protein in Muscovy duckling is 2.98, which is similar to the numbers of *Bombyx mori* (3.07), *Nicotiana tabacum* (3.05), human breast cancer cells (2.71), and human glial cells (2.75) (Fig. 2b)^43–45^.

### Annotation and characterization of the phosphorylated proteins in muscovy duckling

**Figure 3:**
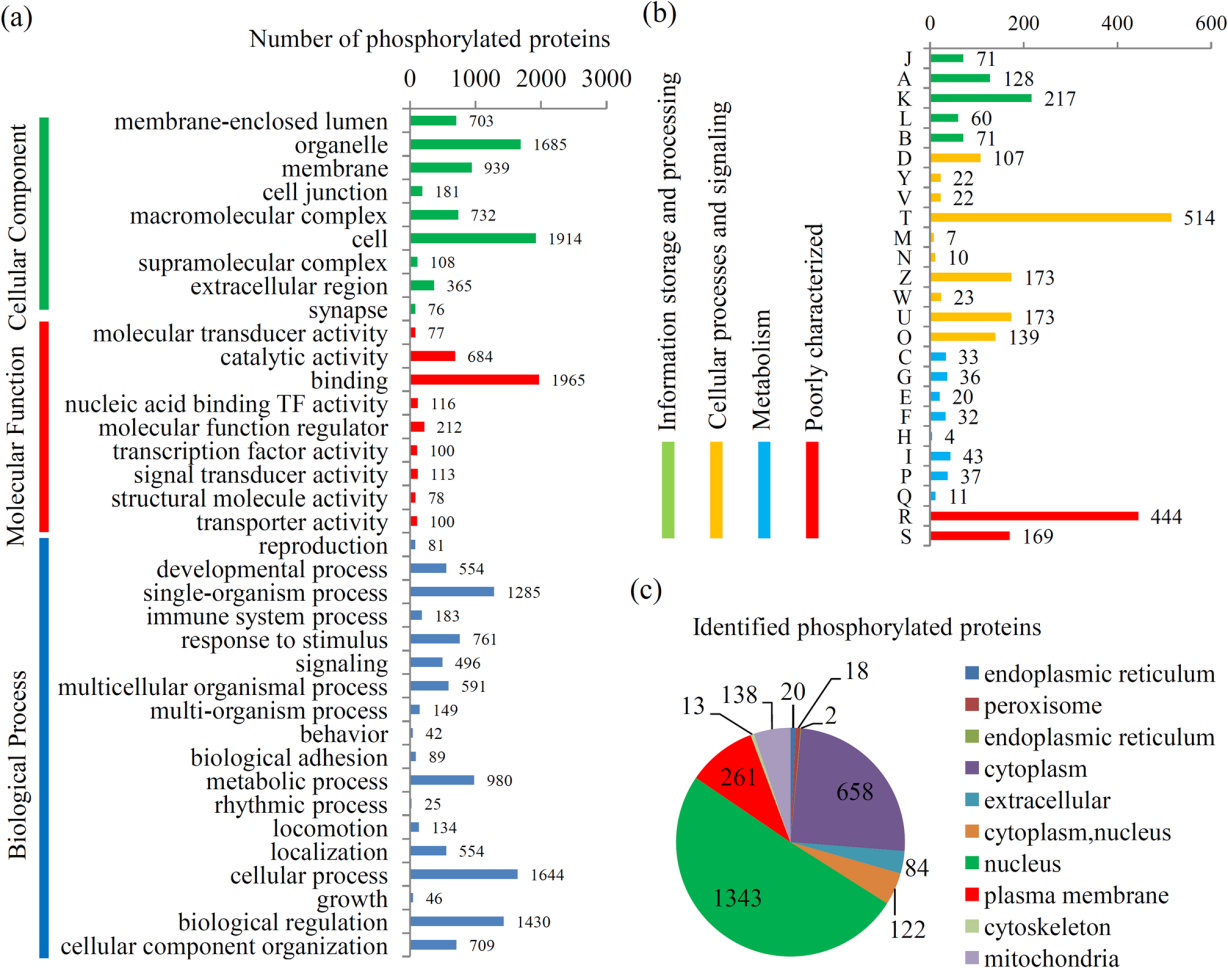
Annotation and classification of phosphorylated proteins. (**a**) GO analysis of all phosphorylated proteins. All proteins were classified by GO terms based on their cellular component, molecular function, and biological process. (**b**) KOG analysis of all phosphorylated proteins. (**c**) Subcellular locations of all phosphorylated proteins.

To predict their possible functions, most of the phosphorylated proteins were classified into three GO categories (Table S2). In the ‘biological process’ category, most of the phosphorylated proteins were classified into the ‘cellular process’ (1,644 proteins) and ‘biological regulation’ (1,430 proteins) terms; in the ‘cellular component’ category, the largest group of phosphorylated proteins were belonged to the ‘cell’ (1,914 proteins) and ‘organelle’ (1,685 proteins) terms; and in the ‘molecular function’ category, the main terms were ‘binding’ (1,965 proteins) and ‘catalytic activity’ (684 proteins) (Fig. 3a).

KOG annotation grouped all of the phosphorylated proteins into four major categories (Table S3). For the ‘information storage and processing’ category, the largest number of phosphorylated proteins were classified into the ‘transcription’ (217 proteins) term; for the ‘cellular processes and signaling’ category, 514 phosphorylated proteins were belonged to the ‘signal transduction mechanisms’ term; for the ‘metabolism’ category, the main terms were ‘lipid transport and metabolism’ (43 proteins) and ‘inorganic ion transport and metabolism’ (37 proteins) (Fig. 3b).

With regard to the subcellular locations, all the identified phosphorylated proteins in Muscovy duckling were grouped into 10 major categories, including 1,343 nucleus-located proteins, 658 cytoplasm-located proteins, 261 plasma membrane-located proteins, and 138 mitochondria-located proteins (Fig. 3c and Table S4).

### Protein motifs associated with phosphorylation

**Figure 4:**
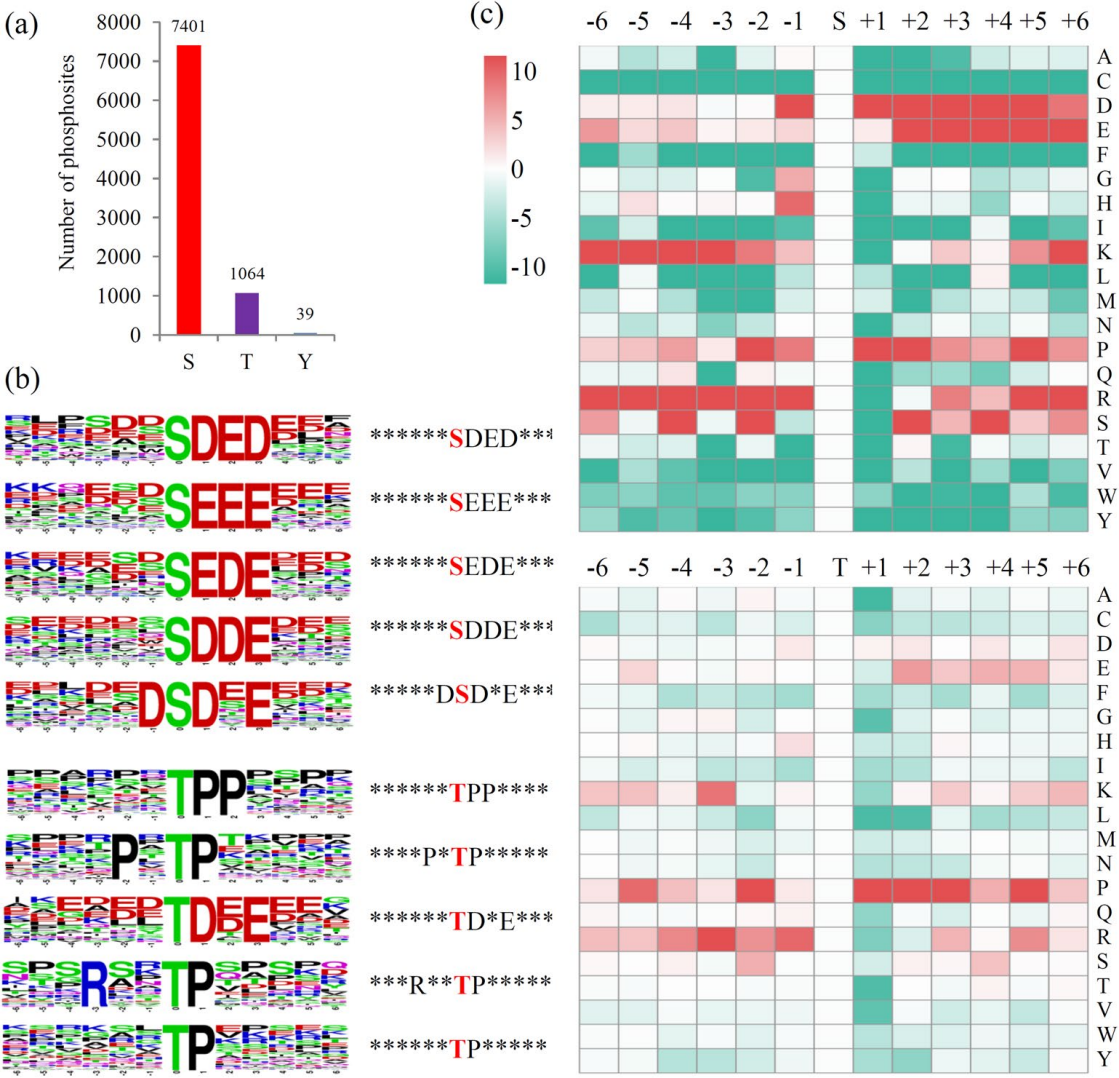
Protein motifs associated with phosphorylation. (**a**) The distribution of phosphorylation sites in different amino acid residues. (**b**) Motif analysis of the amino acids surrounding the phosphorylated residues was performed using MoMo software (V5.0.2 https://meme-suite.org/tools/momo). Sequence logo representation of 5 S-based and 5 T-based conserved phosphorylation motifs. (**c**) A plot showing the relative abundance of amino acids flanking a phosphorylated serine (S) and threonine (T) using the intensity map.

Phosphorylation frequently occurs on three types of amino acid residues, serine (S), threonine (T), and tyrosine (Y) residues^46^. Of all the identified phosphorylation sites in Muscovy duckling, 7,401 sites occur on the S residues, 1,064 sites occur on the T residues, and 39 sites occur on the Y residues (Fig. 4a). To evaluate the nature of the phosphorylated sites, an online tool, Motif-X, was employed to extract overrepresented patterns by comparison with a dynamic background. The results showed that a number of conserved phosphorylation motifs were enriched in the phosphorylated proteins of Muscovy duckling, according to the criteria of a specific sequence spanning six upstream and six downstream residues surrounding each site (Table S5). Five significantly enriched S-based motifs are ‘******SDED***’,‘******SEEE***’, ‘******SEDE***’, ‘******SDDE***’, and ‘*****D**S**D*E***’, and five significantly enriched T-based motifs are ‘******TPP****’, ‘****P*TP*****’, ‘******TD*E***’, ‘***R**TP*****’, and ‘******TP*****’ (Fig. 4b). ‘*’ indicates a random amino acid residue. No Y-based motif was identified.

A position-specific heat map was produced to assess the preference for specific amino acid residues surrounding each phosphorylation site. For the S-based motifs, a strong preference for aspartic acid (D), glutamic acid (E), and proline (P) in the upstream and lysine (K) and arginine (R) in the downstream of the phosphorylation sites were observed. For the T-based motifs, a preference for P in the upstream and P and R in the downstream of the phosphorylation sites were observed (Fig. 4c).

### Differentially phosphorylated proteins (DPPs) responsive to the C/NDRV infections

**Figure 5:**
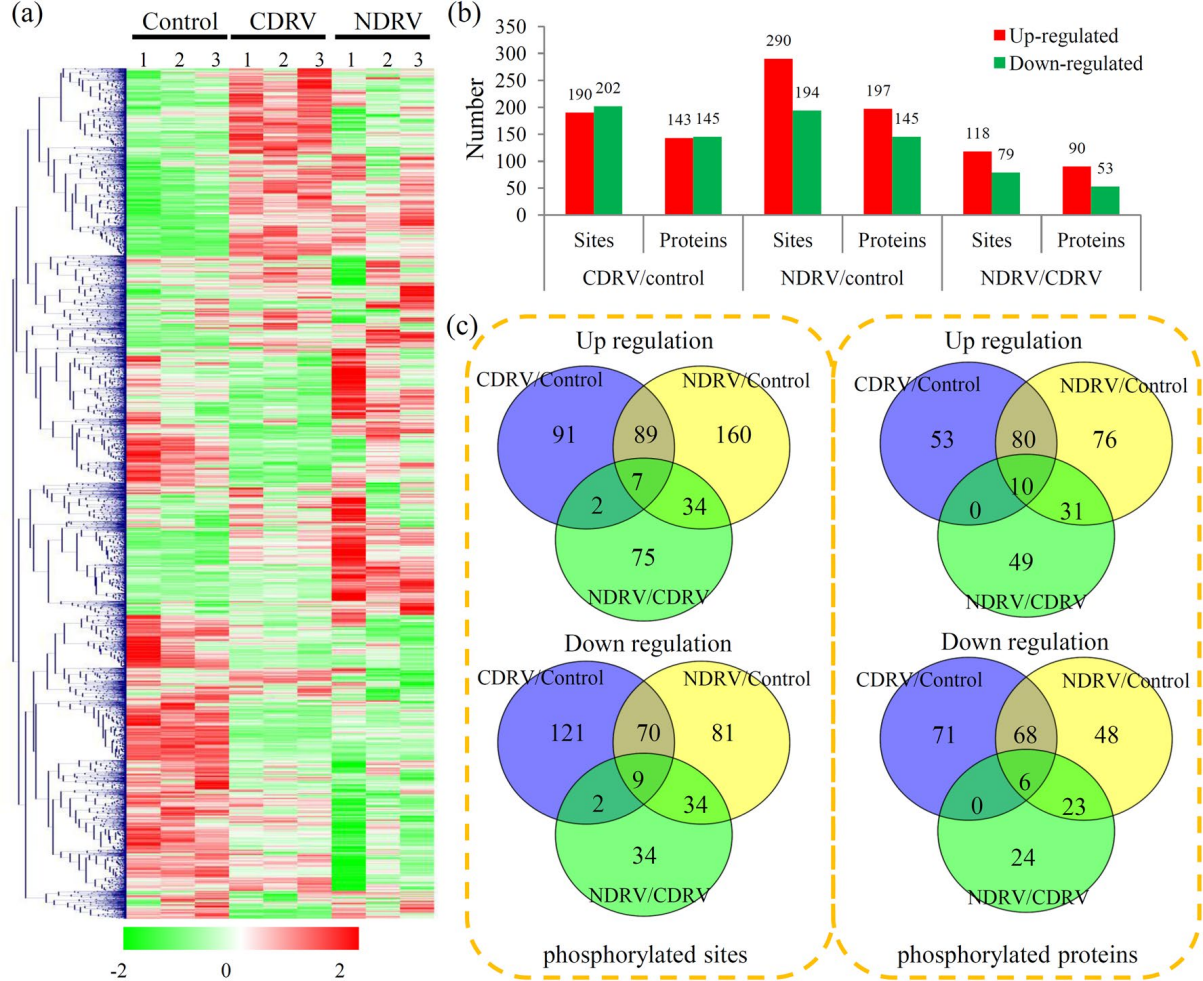
Phosphoproteomic responses of *C. moschata* to C/NDRV infections. (**a**) Heat map for the accumulation levels of all the identified phosphorylated proteins was drawn suing R Package pheatmap (v.2.0.3 https://cran.r-project.org/web/packages/cluster/). Red indicates up-regulation and green indicates down-regulation. The heatmap scale ranges from − 2 to + 2 on a log2 scale. (**b**) The numbers of up- and down-regulated sites and proteins in each comparison. (**c**) Venn diagram showed the numbers of phosphorylation sites and phosphorylated proteins in different comparisons.

After normalization with proteomic data, the effect of differential protein accumulation on the modified signal was removed. Then, the accumulation levels of all phosphorylated proteins in different treatment groups are shown by a heatmap (Fig. 5a). In the CDRV/Control comparison, 190 sites in 143 phosphorylated proteins were up-regulated and 202 sites in 145 phosphorylated proteins were down-regulated (Table S6); in the NDRV/Control comparison, 290 sites in 197 phosphorylated proteins were up-regulated and 194 sites in 145 phosphorylated proteins were down-regulated (Table S7); and in the NDRV/CDRV comparison, 118 sites in 90 phosphorylated proteins were up-regulated and 79 sites in 53 phosphorylated proteins were down-regulated (Fig. 5b and Table S8).

For phosphorylation sites, 96 and 79 sites were up- and down-regulated respectively by both of the C/NDRV infections. For phosphorylated proteins, 90 and 74 proteins were up- and down-regulated respectively by both of the C/NDRV infections (Fig. 5c).

### KEGG enrichment analysis of the DPPs

Under the C/NDRV infections, most of the DPPs were assigned into at least one GO term. In the CDRV/Control comparison, the most significantly enriched ‘biological process’ term was ‘intracellular protein transport’, the most significantly enriched ‘molecular function’ term was ‘rRNA binding’, and the most significantly enriched ‘cellular component’ term was ‘actin cytoskeleton’ (Fig. S1); in the NDRV/Control comparison, the most significantly enriched ‘biological process’ term was ‘intracellular protein transport’, the most significantly enriched ‘molecular function’ term was ‘cell adhesion molecule binding’, and the most significantly enriched ‘cellular component’ term was ‘adherens junction’ (Fig. S2); and in the NDRV/CDRV comparison, the most significantly enriched ‘biological process’ term was ‘posttranscriptional regulation of gene expression’, the most significantly enriched ‘molecular function’ term was ‘G-quadruplex RNA binding’, and the most significantly enriched ‘cellular component’ term was ‘contractile fiber part’ (Fig. S3).

**Figure 6:**
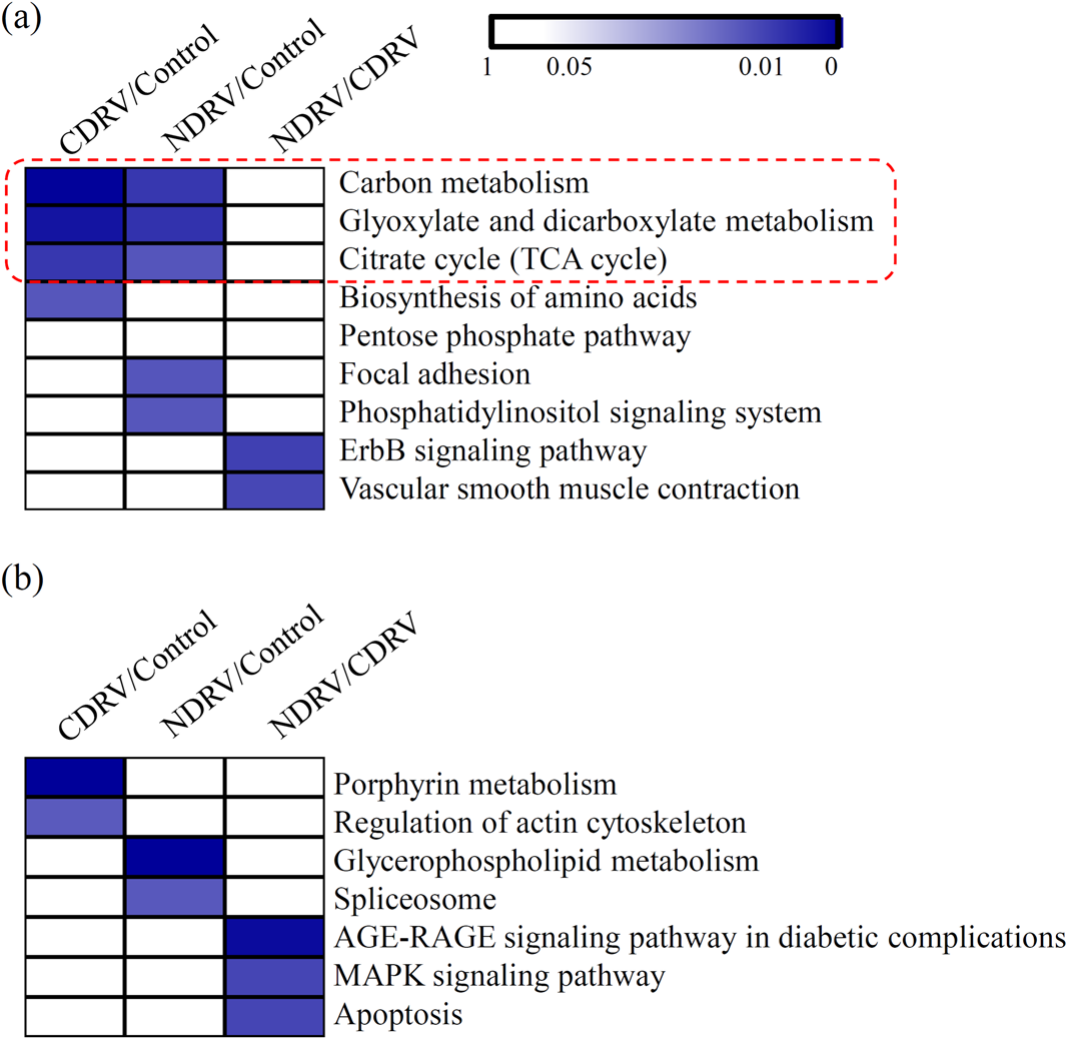
KEGG enrichment analysis of all phosphorylated proteins. (**a**) KEGG enrichment analysis of up-regulated phosphorylated proteins in each comparison. (**b**) KEGG enrichment analysis of down-regulated phosphorylated proteins in each comparison. The significant *P* values of each KEGG term in the three comparisons were shown by heatmaps. The heatmap scale ranges from 0 to 1. All heatmap were drawn suing R Package pheatmap (v.2.0.3 https://cran.r-project.org/web/packages/cluster/).

For the up-regulated DPPs, three important KEGG terms, including ‘carbon metabolism’, ‘glyoxylate and dicarboxylate metabolism’, and ‘citrate cycle’, were significantly enriched in both of the CDRV/Control and NDRV/Control comparisons. The ‘biosynthesis of amino acids’ KEGG term was siginificantly enriched in the CDRV/Control comparison, and the ‘focal adhesion’ and ‘phosphatidylinositol signaling system’ terms were siginificantly enriched in the NDRV/Control comparison (Fig. 6a). For the down-regulated DPPs, the ‘porphyrin metabolism’ and ‘regulation of actin cytoskeleton’ KEGG terms were significantly enriched in the CDRV/Control comparison, and the ‘glycerophospholipid metabolism’ and ‘spliceosome’ KEGG terms were significantly enriched in the NDRV/Control comparison (Fig. 6b).

### Differentially phosphorylated HSPs and serine protease system-related proteins

The roles of HSPs in immunity against virus infections have been deeply investigated^47,48^. In our study, a number of HSPs, including 60 kDa, 71 kDa, 90 kDa, A-, H-, and DnaJ types of HSPs, were identified as phosphorylated proteins (Table 1). The phosphorylation levels of most of the identified HSPs were changed under both of the C/NDRV infections.

**Table 1:** The detail information of Heat shock proteins and proteins involved in serine protease systems.

Protein accession	Position	Amino acid	Protein description	CDRV/control ratio	CDRV/control *P* value	NDRV/control ratio	NDRV/control *P* value	NDRV/CDRV ratio	NDRV/CDRV *P* value
**HSP family**									
R0L1Y3	67	S	Heat shock protein 60 kDa	1.517	0.0061	0.886	0.2014	1.343	0.0307
R0L1Y3	70	S	Heat shock protein 60 kDa	1.506	0.0033	0.973	0.7451	1.465	0.0032
R0L4K0	512	S	Heat shock protein 71 kDa	1.140	0.0507	1.077	0.1689	1.227	0.0019
R0L4K0	178	T	Heat shock protein 71 kDa	1.137	0.3725	1.045	0.6296	1.188	0.1290
R0L4K0	255	S	Heat shock protein 71 kDa	1.013	0.8309	0.772	0.3017	0.782	0.2804
R0L4K0	82	S	Heat shock protein 71 kDa	1.040	0.6768	1.137	0.0455	1.182	0.2111
U3I4J7	723	S	Heat shock protein family A	1.299	0.0837	1.195	0.1878	1.552	0.0055
U3I4J7	43	S	Heat shock protein family A	1.083	0.1397	1.133	0.0485	1.228	0.0265
U3IBT6	777	S	Heat shock protein family H	1.350	0.0123	0.963	0.4622	1.301	0.0106
U3IKZ8	558	S	Heat shock protein family A	1.181	0.0211	1.400	0.0013	1.653	0.0047
U3J1E1	210	S	Heat shock protein 90 family	1.039	0.2883	1.226	0.0392	1.274	0.0173
U3J1E1	669	S	Heat shock protein 90 family	0.875	0.1395	1.209	0.1275	1.057	0.6539
U3I1F6	334	S	DnaJ heat shock protein	1.274	0.0427	0.791	0.0807	1.007	0.9796
U3I7P3	113	S	DnaJ heat shock protein	0.954	0.4859	0.891	0.2783	0.850	0.1415
U3IGK2	381	S	DnaJ heat shock protein	1.374	0.0024	1.245	0.0206	0.906	0.1686
U3J5X4	11	T	DnaJ heat shock protein	1.295	0.2456	1.060	0.7686	1.372	0.2020
U3J5X4	10	S	DnaJ heat shock protein	1.098	0.3506	1.116	0.5984	1.225	0.3447
U3J8M8	588	S	DnaJ heat shock protein	1.129	0.2674	1.119	0.1337	1.054	0.3765
**Serine protease system**									
U3I5A6	392	S	Coagulation factor X	0.586	0.0091	1.6911	0.0041	0.992	0.98
U3I9V9	492	S	Fibrinogen alpha chain	1.263	0.016	0.572	0.0022	0.722	0.013
U3I9V9	462	S	Fibrinogen alpha chain	0.499	0.016	0.674	0.092	0.336	0.01

Two DPPs, including coagulation factor X and fibrinogen α chain, involved in the serine protease systems were identified as phosphorylated proteins (Table 1). There were one phosphorylation site in coagulation factor X (U3I5A6) and two phosphorylation sites in fibrinogen α chain. The phosphorylation level of coagulation factor X was reduced by the CDRV infection and was induced by the DNRV infection. The phosphorylation level of the site S462 of fibrinogen α chain was down-regulated by both of the C/NDRV infections.

### DPPs involved in the innate and adaptive immune responses

**Figure 7:**
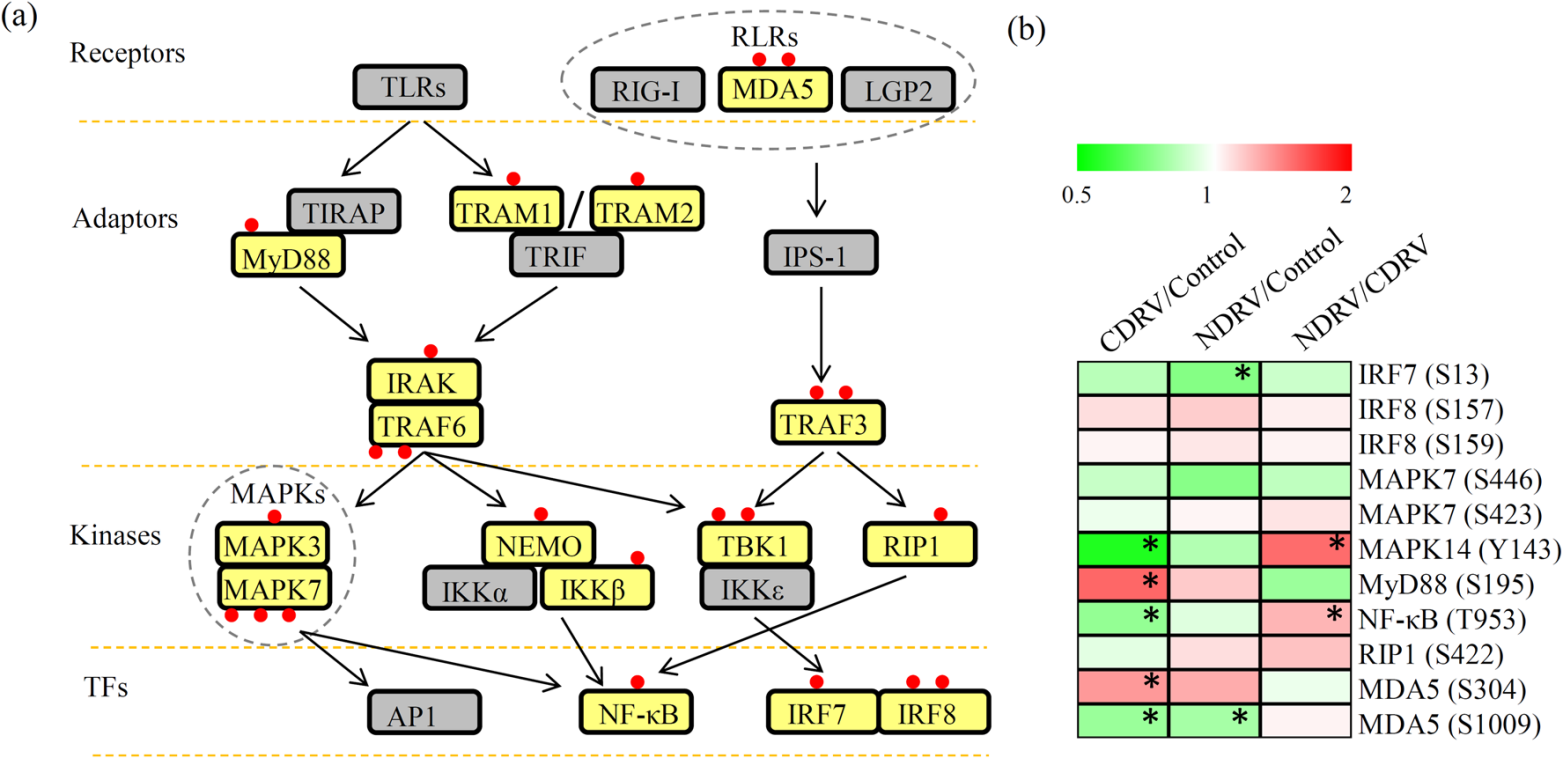
Involvement of innate and adaptive immune responses during the C/NDRV infections. (**a**) Overview of the innate and adaptive immune system in Muscovy duckling. Yellow backgrounds indicate identified phosphorylated proteins. Red dots indicate phosphorylation sites in each phosphorylated protein. (**b**) The accumulation profiles of the proteins involved in innate and adaptive immune system under the C/NDRV infections. The heatmap scale ranges from − 2 to + 2 on a *log*_*2*_ scale. “*” indicate significant differences in each comparison (*P* < 0.05). The heatmap was drawn suing R Package pheatmap (v.2.0.3 https://cran.r-project.org/web/packages/cluster/).

PRRs in innate immune cells play an essential role in first line of host defense system^18^. In our study, 16 proteins involving the intracellular signaling pathways of PRRs were identified as phosphorylated proteins (Table S9). In detail, one RLR type receptor (MDA5), six adaptors (such as TRAM1, TRAM2, MyD88, IRAK, TRAF3 and TRAF6), six kinases (such as MAPK3, MAPK7, NEMO, IKKβ, TBK1 and RIP1), and three TFs (such as NF-κB, IRF7 and IRF8) were identified as phosphorylated proteins. At least one phosphorylation site was identified on each innate system-related phosphorylated protein. Among these proteins, TRAM1, TRAM2, MyD88, IRAK, MAPK3, NEMO, IKKβ, RIP1, NF-κB, and IRF7 contain one phosphorylation site; MDA5, TRAF6, TBK1 and IRF8 contain two phosphorylation sites; and MAPK7 contains three phosphorylation sites (Fig. 7a).

Eleven phosphorylation sites on eight phosphorylated proteins were quantified. In the CDRV/Control comparison, the sites Y143 of MAPK14, T953 of NF-κB and S1009 of MDA5 were siginificantly reduced and the sites S195 of MyD88 and S304 of MDA5 were siginificantly induced. In the NDRV/Control comparison, the sites S13 of IRF7 and S1009 of MDA5 were siginificantly down-regulated. In the NDRV/CDRV comparison, the sites Y143 of MAPK14 and T953 of NF-κB were siginificantly increased (Fig. 7b).

## Discussion

Duck is a major waterfowl species with a population of up to 30 billion per year in China^21^. C/NDRV is fatal pathogens that endanger ducks and cause a large loss in the Chinese poultry industry^49^. PTMs, phosphorylation in particular, are ubiquitous strategy for the host responses to virus infections^37^. Therefore, it is of great significance to investigate the responses of PTM to the C/NDRV infections.

Protein phosphorylation is a universal PTM; however, the abundance of phosphorylation largely varies among different species. In *C. moschata*, the abundance of phosphorylated protein (2,853 phosphorylated proteins) was higher than most of the published species, such as *Sus domesticus* (966 phosphorylated proteins), *Bombyx mori* (2,112 phosphorylated proteins), *Kandelia candel* (1,516 phosphorylated proteins), *Nicotiana tabacum* (1,311 phosphorylated proteins), *Lotus japonicus* (1,154 phosphorylated proteins), *Ammopiptanthus mongolicus* (2,019 phosphorylated proteins), *Catalpa fargesii* (1,646 phosphorylated proteins), *Abelmoschus esculentus* (2,550 phosphorylated proteins), and *Physcomitrella patens* (1,873 phosphorylated proteins)^35,43,45,50–53^. High-throughput method gives us an opportunity to screen potential phosphorylated proteins and reveal the involvement of protein phosphorylation in the responses to the C/NDRV infections. The number of phosphorylation sites per protein is near 3, higher than most of the published phosphoproteomes, indicating a high degree phosphorylation level of *C. moschata* proteome.

Infectious bursal disease virus infection siginificantly affected the energy metabolisms^54^. Several intermediates of the TCA cycle, which generates ATP to provide energy for key cellular functions, are involved in the innate immune system during infection and inflammation^55^. In our study, enrichment analysis showed that phosphorylation levels of the enzymes involved in the TCA cycle was significantly up-regulated by both of CDRV and NDRV infections. It suggested that higher phosphorylation level of the TCA cycle might provide more energy to enhance the responses to the C/NDRV infections. Moreover, 143 DPPs were identified in the NDRV/CDRV comparison, suggesting a great difference between the classic and novel DRVs. The ErbB signaling pathway and MAPK signaling pathway participate in regulating diverse biologic responses, including proliferation, differentiation, cell motility, senescence, apoptosis and survival^56,57^. KEGG enrichment analysis showed that the ErbB signaling pathway was significantly up-regulated and the MAPK signaling pathway was significantly down-regulated in the NDRV/CDRV comparison. Changes in the phosphorylation levels of the two disease-related signaling pathways might be required for the responses to the C/NDRV infections.

Our previous works indicated a central role of the network of seine proteases, consisting of the complement, coagulation and fibrinolytic systems, in the innate immune reactions^28,29^. However, the roles of protein phosphorylation in the responses of serine protease system to the C/NDRV infections are largely unknown. Under virus infection, coagulation cascade is activated to fight against the invasion of pathogenic microorganisms^58^. In the coagulation cascade, coagulation factor X is a plasma serine endopeptidase that plays a chief role in blood coagulation^59^. In the present study, coagulation factor X was identified as phosphorylated protein, and its phosphorylation site (S393) was significantly reduced by the CDRV infection and induced by the NDRV infection. It suggested that phosphorylation of the S393 site in coagulation factor X might be involved in the blood coagulation pathway of *C. moschata*. Fibrinogen, a soluble protein with three chains, including α, β and γ chains, is a therapeutic target for bleeding^60^. Two phosphorylation sites in the α chain of fibrinogen were identified in *C. moschata*, and one (S492) of which was significantly down-regulated by the CDRV infection and up-regulated by the NDRV infection. Changes in phosphorylation level of fibrinogen might affect the control of blood coagulation under the C/NDRV infections. Though the importance of complement proteins in serine protease system is well known, no phosphorylation site was detected in complement proteins.

Chaperones belong to a class of small compounds and play essential roles in the protein folding process^61^. Chaperones, HSPs in particular, participate in activation of the immune system^62^. In our study, 11 HSPs were identified as phosphorylated proteins, such a 71 kDa HSP with four phosphorylation sites, a 60 kDa HSP with two phosphorylation sites. Increasingly, several phosphorylation sites of the 60 kDa HSPs, such as the S67 and S70 of 60 kDa HSP, were up-regulated under the CDRV infection, suggesting a role of phosphorylation in the folding of immunologically important proteins.

To effectively respond to environmental antigens, the immune system is exquisitely balanced^63^. PTMs, such as phosphorylation, help to ensure the balance of immune signal transduction by activating or terminating the immune responses at the proper time^64^. In our study, GO analysis showed that 183 phosphorylated proteins belonging to the ‘immune system process’ term. In detail, 16 phosphorylated proteins involving the signaling pathways of PRRs, including receptors, adaptors, kinases and downstream TFs, were identified, suggesting a crucial role of protein phosphorylation in host immune responses of *C. moschata*.

Recognition of pathogen-associated molecular patterns by PRRs is the initial step of innate and adaptive immune responses^16^. RIG-I-like receptors, including RIG-I, MDA5 and LGP2, are the primary sensors for recognizing viral RNA^65^. MDA5 functions as a cytoplasmic double-stranded RNA sensor and plays a critical role in antiviral innate immunity. PTMs are essential for activation of MDA5^18^. For example, ubiquitination of MDA5 at lysine 743 is critical for oligomerization and activation of MDA5^66^. In *C. moschata*, two phosphorylation sites (S304 and S1009) were identified in MDA5, suggesting that phosphorylation of MDA5 at serine 304 and 1,009 might also be required for the activation of MDA5. Adaptor molecules act as messengers that deliver signals from the receptors to activate various downstream kinases and TFs. MyD88, a well characterized adaptors in TLR signaling, is located in the cytosol near the cytosolic part of TLRs and delivers an activation signal to activate NF-κB^67^. In *C. moschata*, there is one phosphorylation site (S195) in MyD88, and its phosphorylation level was significantly up-regulated under the CDRV infection. Phosphorylation of MyD88 might enhance the activation signal that is initiated by receptor activation. Further, another five adaptors, such as TRAM1/2, IRAK, TRAF3/6, were identified as phosphorylated proteins, although no quantitative data was available.

A series of kinases that was activated by pathogen signals from adaptors can phosphorylate downstream TFs^18^. For examples, IκB kinase complex, consisting of two catalytic components, IKKα and IKKβ, and a regulatory component, NF-κB essential modifier (NEMO), acts as main kinase^21,68^. TRAF family member-associated NF-κB activator binding kinase-1(TBK1) functions as a kinase in the phosphorylation and activation of IFN regulatory factor3 (IRF3)^68,69^. In our study, NEMO, IKKβ, and TBK1 contained one phosphorylation each, suggesting that phosphorylation might be involved in their function of kinase. Phosphorylated IκB is degradated by ubiquitination^18^. In *C. moschata*, phosphorylated IKKβ might be responsible for the degradation of IκB kinase complex.

A number of TFs have been previously reported in PRRs. As downstream TFs, NF-κB, AP1, and IRF produced effecter molecules including cytokines, chemokines, inflammatory enzymes, and type I interferones^70^. Our results showed that the phosphorylation levels of NF-κB (T953) and IRF7 (S13) were siginificantly reduced under CDRV and NDRV infections, respectively. Further analysis of how these phosphorylated TFs contributes to the responses to the C/NDRV infections is needed.

Recently, the roles of protein phosphorylation in host cell response to virus infection have been reported. For example, phosphorylation of AKT-mTORC1 pathway led to disruption of DNA double-strand breaks-induced herpes simplex virus-1 reactivation^71^. In ducks, disruption of C/NDRV induced host protein phosphorylation might contribute to virus control.

Using a high-resolution LC–MS/MS integrated to highly sensitive immune-affinity antibody method, phosphoproteomes of *Cairna moschata* spleen tissues under the C/NDRV infections were analyzed. A total of 8,504 phosphorylation sites were identified on 2,853 proteins, of which 6,343 sites of 2,407 proteins were quantified. The phosphorylation levels of coagulation factor X and fibrinogen α chain were changed, suggesting an enhancement of blood coagulation under the C/NDRV infections. Furthermore, 16 proteins involving the intracellular signaling pathways of PRRs were identified as phosphorylated proteins, suggesting that phosphorylation might also be required for the activation of innate and adaptive immune responses. Our study provides new insights into the responses of ducklings to the C/NDRV infections at the PTM level.

## Supplementary information

Supplementary Information 1.

Supplementary Information 2.
