# Supplementary material for: The phosphoproteomic responses of duck (Cairna moschata) to classical/novel duck reovirus infections in the spleen tissue
Source: Sci Rep. 2020 Sep 17;10:15315. doi: 10.1038/s41598-020-72311-0 (PMC7499213; doi:10.1038/s41598-020-72311-0)
Supplement: Supplementary file 2 — Supplementary Information 2. [file 41598_2020_72311_MOESM2_ESM.pdf]

The phosphoproteomic responses of duck (*Cairna moschata*) to classical/novel duck reovirus infections in the spleen tissue

Tao Yun, Jionggang Hua, Weicheng Ye, Zheng Ni, Liu Chen, Cun Zhang *

Institute of Animal Husbandry and Veterinary Sciences, Zhejiang Academy of Agricultural Sciences, Hangzhou 310021, China

* Corresponding author:

Cun Zhang

Email Address: zhangcun@aliyun.com

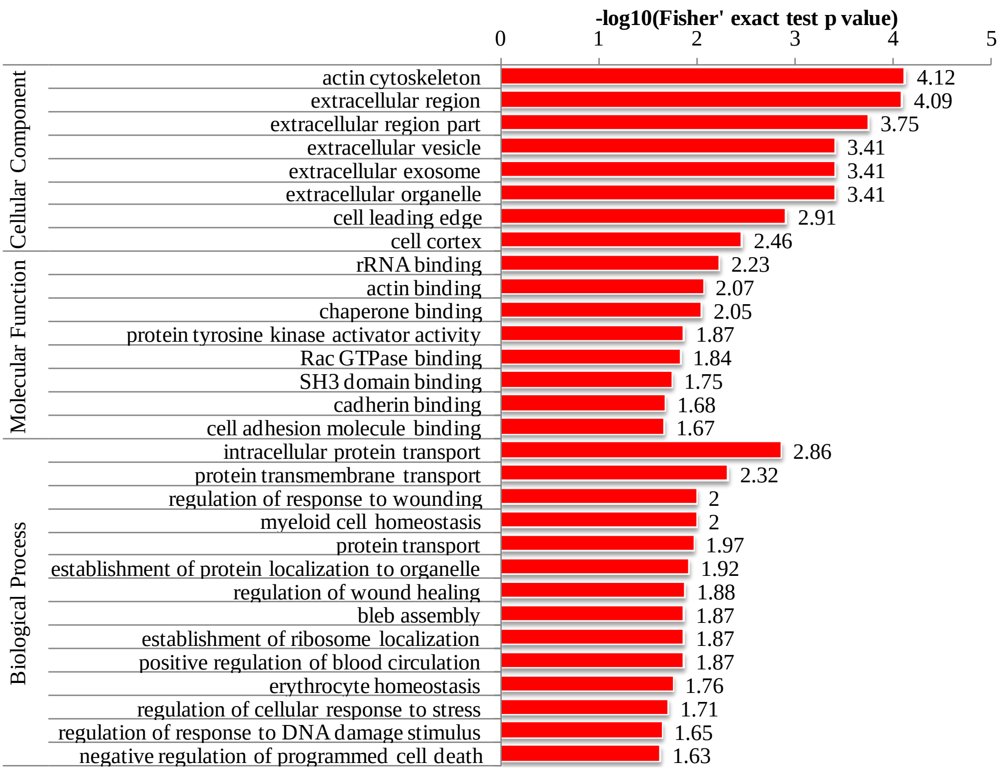

Figure S1 **KEGG enrichment analysis of the DPPs in the CDRV/Control comparison.**

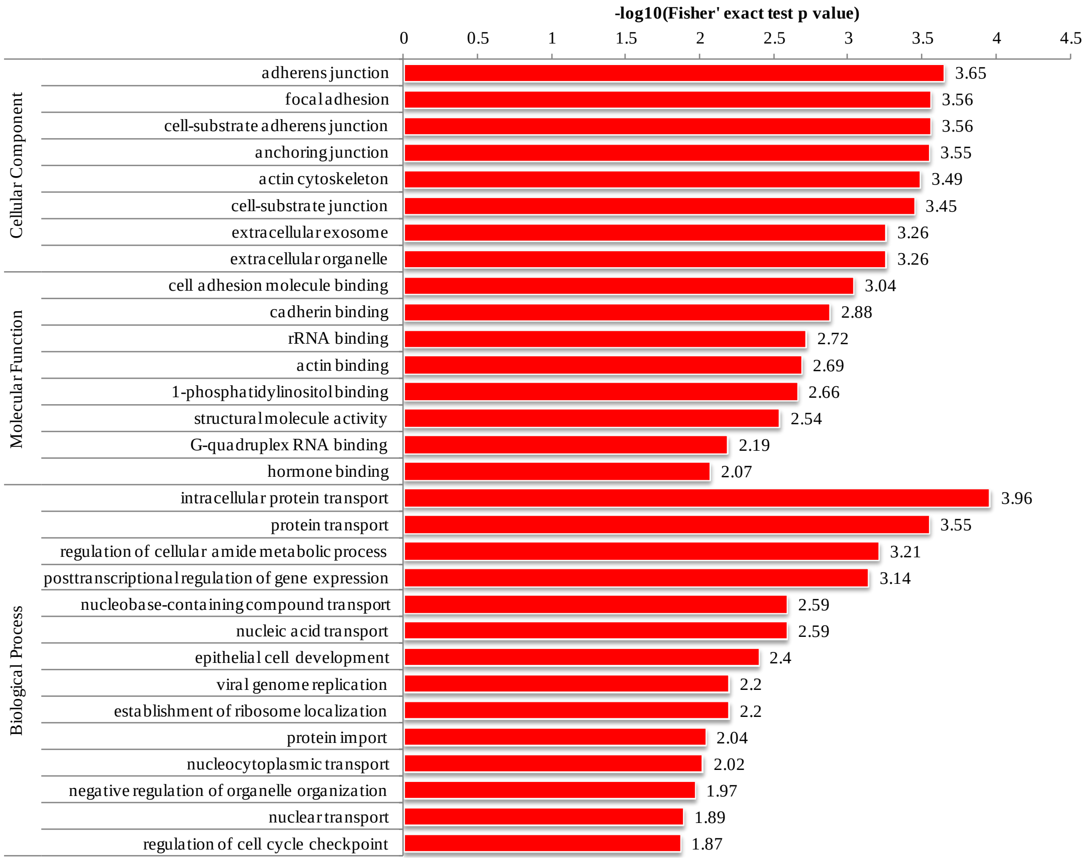

Figure S2 **KEGG enrichment analysis of the DPPs in the NDRV/Control comparison.**

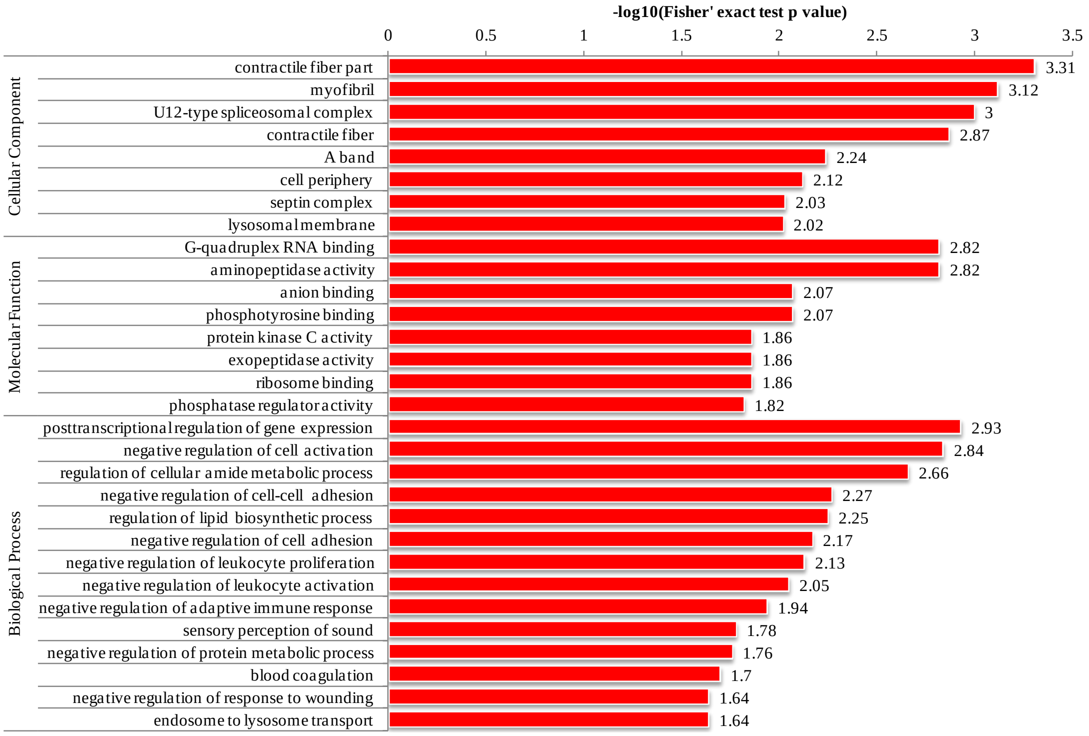

Figure S3 **KEGG enrichment analysis of the DPPs in the NDRV/CDRV comparison.**
